# Case Report: Autosomal recessive palmoplantar keratoderma with additional bilateral hearing loss due to a pathogenic frameshift deletion in *FAM83G*

**DOI:** 10.3389/fmed.2025.1687811

**Published:** 2025-11-26

**Authors:** Mónica Mora-Gómez, Marta Feito, Natalia Gallego-Zazo, Rocío Maseda-Pedrero, Tristán G. Sobral-Costas, Lucía Miranda-Alcaraz, Valeria Vásquez-Amell, Manuel Rodríguez-Canó, Alejandro Parra, Mario Cazalla, Pedro Arias, Cristina Silván, Juan A. Jiménez-Estrada, Víctor L. Ruiz-Pérez, Julián Nevado, Pablo Lapunzina, Jair Tenorio-Castano

**Affiliations:** 1 CIBERER, Centro de Investigación Biomédica en Red de Enfermedades Raras, Madrid, Spain; 2 INGEMM-IdIPAZ, Institute of Medical and Molecular Genetics, La Paz University Hospital, Madrid, Spain; 3 ITHACA, European Reference Network, Brussels, Belgium; 4 Dermatology Unit, La Paz University Hospital, Madrid, Spain; 5 Instituto de Investigaciones Biomedicas Sols-Morreale (IIBM), CSIC-UAM, Madrid, Spain

**Keywords:** palmoplantar keratoderma, *FAM83G*, genodermatosis, skin disorder, bilateral hearing loss, genomic medicine, massive paralleled sequencing

## Abstract

Palmoplantar keratoderma (PPK) comprises a group of genodermatosis disorders phenotypically characterized by the isolated thickening of the skin of palms and soles. Syndromic forms can also include other phenotypic features in addition to those affecting the skin. Genetics plays a major role in the etiology and classification of PPK, particularly in syndromic cases, although the genetic mechanisms underlying some cases remain largely unknown. Here, we present a patient from a consanguineous family in which a homozygous variant was identified through whole exome sequencing in the *FAM83G* gene. The identified variant consists of the deletion of one nucleotide and a subsequent frameshift, leading to an early stop codon and a potentially truncated protein. *FAM83G* gene has been associated with PPK relatively recently, and therefore, the phenotype arising from mutations in this gene needs further refinement based on the small number of reported cases. The phenotype of the patient included keratoderma both in hands and feet and bilateral hearing loss, without hair or tooth abnormalities. This patient adds new clinical features and molecular supporting information for this novel genodermatosis syndrome with an apparently autosomal recessive pattern of inheritance. This entity caused by *FAM83G* pathogenic variants can be named as FAM83G-associated palmoplantar keratoderma.

## 1 Introduction

Palmoplantar keratoderma (PPK) comprises a heterogeneous group of acquired and hereditary disorders characterized by hyperkeratotic lesions on the surface of palms and soles (1, 2). Hereditary forms of PPK can be subdivided into non-syndromic (only isolated hyperkeratosis) and syndromic forms (2). The latter can be associated with cutaneous and non-cutaneous features in other organs apart from the skin, such as hearing loss, cardio-myopathies, carcinoma and benign tumors (2, 3).

The classification of keratodermas mainly depends on whether the disease has been inherited or acquired, and whether it is isolated or syndromic. There are three large groups of keratodermas: (A) diffuse keratodermas that in general only affects the palms and soles, (B) focal keratodermas mainly affecting pressure areas and (C) punctate-type keratodermas that can result in tiny bumps of the hands and soles (4). In terms of molecular mechanisms, both autosomal dominant and recessive inheritance have been described in PPK and about 72 genes have been associated with this condition, 61 of them with high level of evidence (5).

Among the genes implicated in PPK, pathogenic variants in *KRT1*, *KRT9*, and *KRT16*, which encode keratins, are well-documented. These keratins are essential for the structural integrity of the epidermis and the resilience of the skin under mechanical stress (6). In addition to pathogenic variants in keratin genes, other genes such as *GJB2*, which encodes connexin 26, and *SERPINB7*, which is involved in cellular stress responses, have been associated with specific subtypes of PPK (7, 8).

Treatment of patients with PPK primarily aims to improve skin thickening, reduce pain, and alleviate functional impairment (9). Common treatments include topical emollients, keratolytic agents, topical or oral retinoids, and topical vitamin D; however, there is currently no cure for this disease. Overall, oral retinoids appear to be the most effective treatment for PPK, but their efficacy depends on the subtype of the disease.

The *FAM83G* gene, located on chromosome 17p11.2, encodes a protein composed by 823 amino acids (10). This protein, also known as PAWS1 (Protein Associated with SMAD 1), belongs to the FAM83 protein family sharing the conserved DUF1669 (Domain of Unknown Function) domain at the N-terminus (11). The FAM83 protein family plays a crucial role in cellular signaling pathways. Specifically, FAM83G has been shown to interact with components of the PI3K-AKT signaling pathway, which is integral to many cellular processes, and its dysregulation can lead to abnormal keratinocyte proliferation, contributing to the hyperkeratotic phenotype observed in PPK (12, 13). FAM83G is also part of a complex that mediates ẞ-catenin degradation, a process inhibited via Wnt signaling pathway (1). In addition, this protein can interact with BMP receptors modulating their signaling activity (14). Both Wnt and BMP signaling pathways have been linked to the development of the skin and hair follicles (15, 16).

Here we report a 33-year-old Algerian women diagnosed with PPK, presenting with a likely pathogenic homozygous variant in *FAM83G* and the common core clinical features, along with additional characteristics.

## 2 Case presentation

**Figure 1:**
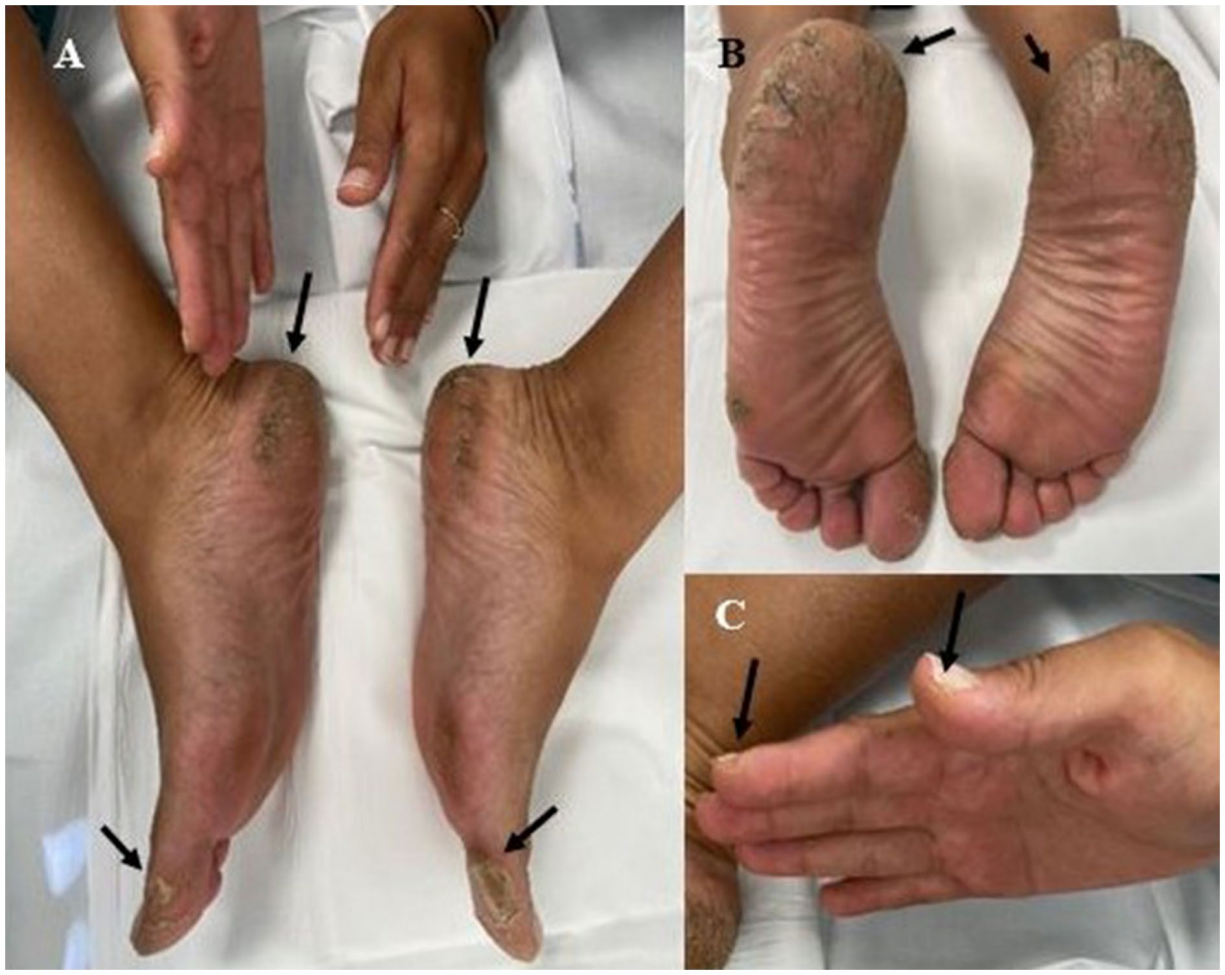
Skin phenotype of patient with *FAM83G* homozygous variant. **(A–C)** Focal palmoplantar keratoderma. Areas of palmoplantar skin under pressure are disproportionally thickened (black arrows).

The patient, a woman aged 33 years from a consanguineous family, presents PPK in palms and soles and bilateral hearing loss with the onset of palmoplantar hyperkeratosis at 2 years of age, approximately. She showed marked hyperkeratosis in heels and the lateral face of both soles, as well as weight-bearing areas. In hands, lesions are mainly located in the fingertips (Figure 1). There is no evidence of transgradiens involvement, as the lesions do not extend to the top of the hands and feet. No abnormalities in teeth or hair were observed in the patient. She also presents bilateral hearing loss, and recently, she was confirmed with the diagnosis with moderate–severe sensorineural hearing loss predominantly in high frequencies, bilateral. Thus, in 2016 she underwent tympanoplasty with closed mastoidectomy of the left ear with autologous fascia graft using a medial technique.

Therapeutic approaches for *FAM83G*-associated palmoplantar keratoderma (PPK) remain largely symptomatic, as there are currently no targeted molecular therapies described in the literature for this condition, as far as we know. Regarding the patient’s treatment, it includes the topical application of Vaselix 40 on the affected areas. This medication contains 40% urea and is used to soften hyperkeratotic skin, promote exfoliation, and improve hydration in thickened or scaly lesions.

**Figure 2:**
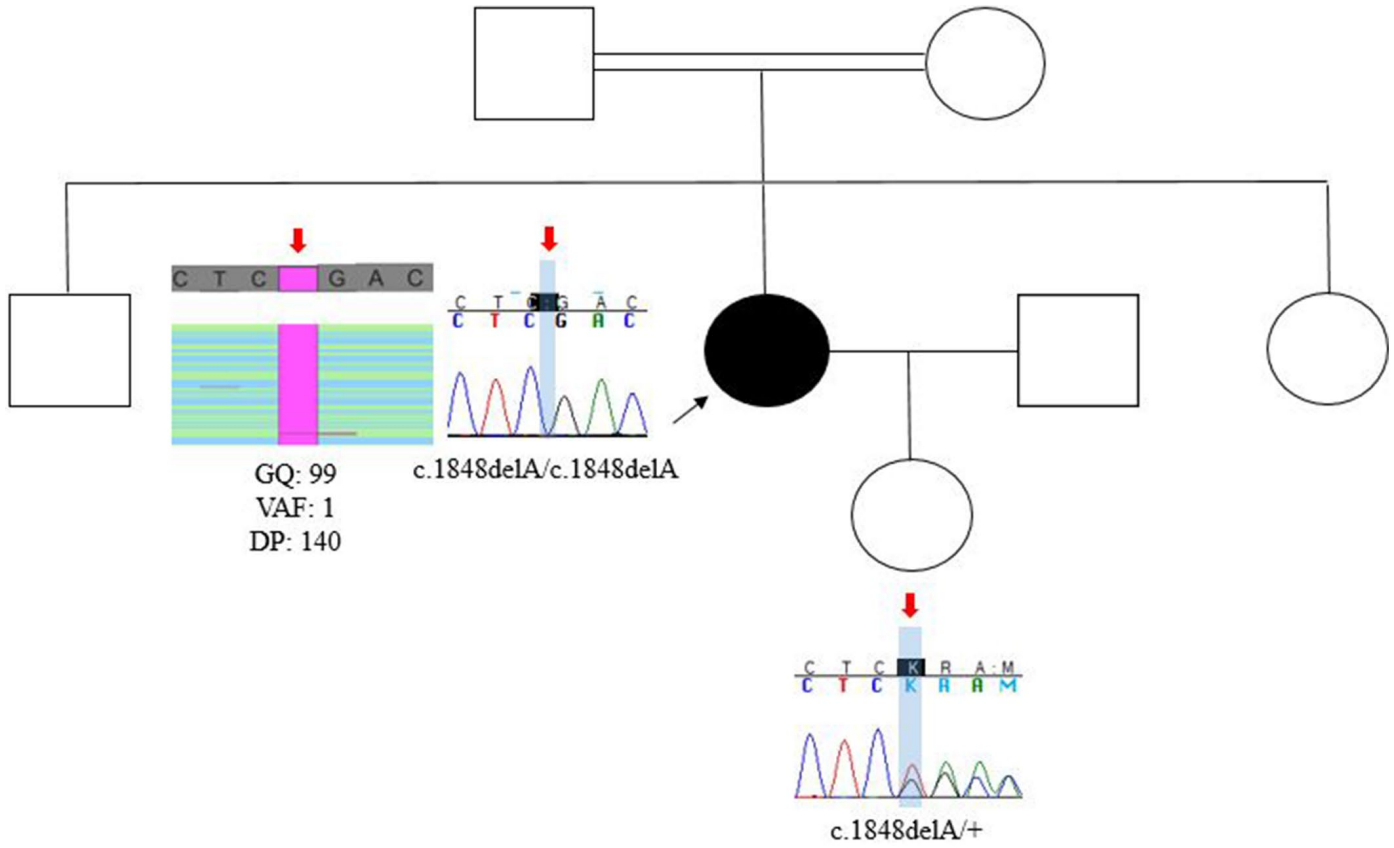
Pedigree and segregation analysis in available familiar cases. The proband is the middle of three siblings and the unique affected member of a consanguineous family. Segregation analysis con-firmed that the variant is present in the proband in the homozygous state and her daughter is a heterozygous healthy carrier (red arrows). DP, read depths; GQ, genotype qualities; VAF, variant allele fraction.

She is the middle daughter among three siblings. Neither her parents nor her siblings have similar alterations. Additionally, she is the mother of a 3-year-old girl who is unaffected (Figure 2).

**Figure 3:**
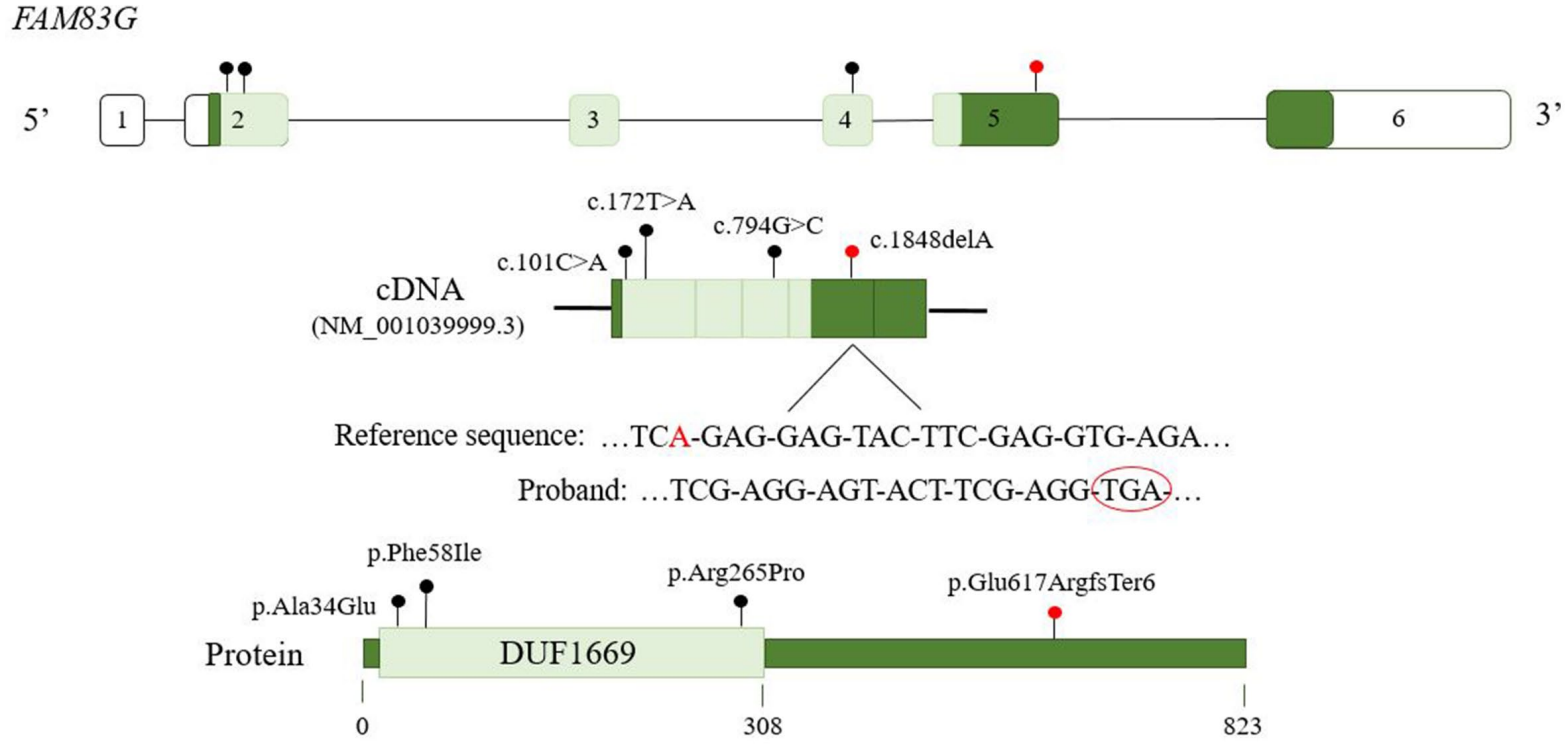
*FAM83G* gene, protein domain structure and location of the variants identified in association with the development of PPK. The variant (NM_001039999.3:c.1848delA) identified in our patient (labelled in red) causes the deletion of one nucleotide and the appearance of an early stop codon. Variants c.101C > A (p.Ala34Glu) and c.794G > C (p.Arg265Pro) were reported in Maruthappu et al. (3) and Glennie et al. (24). Variant c.172 T > A (p.Phe58Ile) was reported in van Gisbergen et al. (25), that described another variant causing the complete deletion of the gene.

Whole exome sequencing (WES) analysis allowed the identification of a likely pathogenic homozygous variant in exon 5 of *FAM83G*: [chr17:18881131, [hg19]; NM_001039999.3:c.1848delA:p.(Glu617Argfs*6)]. Segregation analysis by Sanger sequencing confirmed that the variant is present in homozygous state in the proband, while her daughter is a heterozygous healthy carrier (Figure 2). Genetic testing in the parents of the proband was not available since they live in another country. The variant is predicted to cause the deletion of one nucleotide and the generation of a premature stop codon (Figure 3).

## 3 Discussion

PPK encompasses a heterogeneous group of genodermatosis disorders primarily characterized by abnormal thickening of the skin on the palms and soles. The clinical presentation of PPK can vary significantly, ranging from diffuse thickening to focal or punctate lesions. These conditions are often associated with pain and secondary infections and can significantly impair the quality of life of affected individuals. The pathogenesis of PPK is complex, involving both genetic and environmental factors (17–19).

PPK can be inherited in autosomal dominant, autosomal recessive, or X-linked pat-terns, with pathogenic mutation identified in more than 70 different genes. These genetic variants can disrupt various biological pathways that regulate keratinocyte proliferation and differentiation, leading to the characteristic hyperkeratosis seen in PPK (17–19).

We report a 33-year-old women from a consanguineous family presenting with a homozygous loss-of-function *FAM83G* variant associated with PPK. The phenotype of the patient included palmoplantar keratoderma, thickening of the skin on soles and bilateral hearing loss (Figure 1). There has been no previous report including bilateral hearing loss as part of the clinical features of FAM83G syndrome, so presently we cannot discern whether this is an infrequent finding of the disorder or it has a different etiology not related to the disease, since bilateral hearing loss may be due to other non-genetic causes such as infections. Conversely, expression of this gene has not been described in regions related to the ear, such as the cochlea, nor in cells involved in the hearing process according to The Human Protein Atlas (20). However, whole exome sequencing did not reveal any other variant in the proband that could be associated with the bilateral hearing loss, including the analysis of the entire coding region and intron–exon boundaries of *GJB2*, which was initially suspected as the causative gene in this patient. Also, a genetic testing carried out in 2020, discard the presence of a variant in a panel of genes associated with congenital hearing loss.

The homozygous variant detected in the patient causes the deletion of one nucleotide within exon 5 of the gene, leading to a frameshift and the generation of a stop codon six amino acids downstream from the variant that results in a truncated protein (Figure 3). The aberrant transcript is predicted to be degraded through the nonsense-mediated decay (NMD) machinery (21). Additionally, the detected variant has only been reported in gnomAD exomes v4.1 in low frequency (0.00000069) in heterozygous state, but not in other pseudocontrol population databases analyzed (gnomAD genomes v4.1, 1000G, ESP, Kaviar, Beacon, Bravo). The variant presents a frequency of 0.00000138 in men, but it has not been detected in women. Reviewing gnomAD v4.1, 115 pLoF variants have been reported, being all except two in heterozygous state, with a pLoF score for intolerance to loss of function variants of 0.86 which predicts that the gene is intolerant to pLoF changes, adding evidence for the pathogenicity of the variant detected.

Since the variant was found in the homozygous state and the parents of the proband are consanguineous, we suggest that the parents must be obligated carriers. However, segregation analysis could not be performed, as the parents were unavailable for genetic testing at the time of this report due to residing abroad. Additionally, segregation analysis in her unaffected daughter confirmed that she is a healthy carrier of the deletion (Figure 2), which should be considered for reproductive genetic counseling in the future.

In 2014, a study reported a homozygous missense *FAM83G* variant in dogs with hereditary footpad hyperkeratosis (HFH), characterized by cracked surfaces and deep fissures of the foot pads and woolly hair (22). In a mouse model of woolly hair, it was observed that the underlying defect associated with the phenotype is a deletion encompassing *FAM83G* (23).

More recently, PPK has been related to an autosomal recessive inheritance of variants in the *FAM83G* gene. Four cases with PPK and variants in *FAM83G* gene have been reported so far, all of them from consanguineous families (3, 24, 25). One report described two siblings with PPK affecting the soles, dystrophic toenails and thick, bushy hair (3). Additionally, three more cases were reported in 2024 (24, 25). One describe a woman with affected skin on her hands and feet, dystrophic nails, affected dental enamel and thin, curly hair (24). In both cases, a two different homozygous missense pathogenic variant in *FAM83G* were detected [c.101C > A:p.(Ala34Glu) and c.794G > C: p.(Arg265Pro)] (3, 24). The other two cases were a boy diagnosed at 3 years of age and a 26-year-old woman. The boy present diffused PPK in heels and painful fissures especially on the pressure points. In addition, he has hyperkeratosis in fingertips, dystrophic nails and mild keratotic plaques and keratosis pilaris in knees and elbows. In this case, a missense pathogenic variant in homozygous state was also reported [c.172 T > A p.(Phe58Ile)]. On the other hand, the 26-year-old woman, who presented the first symptoms at 2 years of age, has hyperkeratosis in fingertips and soles with painful fissures and dystrophic finger and toenails. In this case, they identified a homozygous deletion of *FAM83G* gene [NM_001039999.3:c.(?_-184)_(*2570_?)del] (25).

The protein encoded by *FAM83G* plays an important role in Wnt signaling, which regulates the early development of skin (1). Functional studies involving the Ala34Glu PPK variant, including the knock-in of this variant in a human cell line, showed an alteration of canonical Wnt signaling. These studies also suggested a decreased protein stability of Ala34Glu variant in comparison with the wild type protein (11). Additionally, the FAM83G protein can modulate BMP signaling, which is essential for the control of cell differentiation and apoptosis in the developing epidermis and hair follicles (14). FAM83G has also been associated with the PI3K-AKT signaling and thus pathogenic variants in *FAM83G* may lead to alterations of this pathway, disrupting normal cellular homeostasis and promoting the pathological keratinocyte behavior seen in PPK (12).

The exact mechanism by which *FAM83G* pathogenic variants contribute to PPK is still under investigation, but current evidence suggests that FAM83G variants may have an impact in activity of various key signaling pathways in the affected tissues. Considering the functional implications of FAM83G and the previously described cases, it is plausible that the loss of function of FAM83G generates an alteration of these pathways responsible for the appearance of PPK in the patient.

In summary, our findings provide further evidence of the association between pathogenic variants in *FAM83G* and the development of PPK, and we propose that this entity may be named *FAM83G*-associated palmoplantar keratoderma syndrome according to the dyadic approach guidelines proposed (26).

## Data Availability

The datasets presented in this study can be found in online repositories. The names of the repository/repositories and accession number(s) can be found at: https://www.lovd.nl/3.0/home, variant ID: 0001047221.
