# Correction: Case Report: Autosomal recessive palmoplantar keratoderma with additional bilateral hearing loss due to a pathogenic frameshift deletion in FAM83G

**DOI:** 10.3389/fmed.2026.1779531

**Published:** 2026-02-13

**Authors:** Mónica Mora-Gómez, Marta Feito, Natalia Gallego-Zazo, Rocío Maseda-Pedrero, Tristán G. Sobral-Costas, Lucía Miranda-Alcaraz, Valeria Vásquez-Amell, Manuel Rodríguez-Canó, Alejandro Parra, Mario Cazalla, Pedro Arias, Cristina Silván, Juan A. Jiménez-Estrada, Víctor L. Ruiz-Pérez, Julián Nevado, Pablo Lapunzina, Jair Tenorio-Castano

**Affiliations:** 1 CIBERER, Centro de Investigación Biomédica en Red de Enfermedades Raras, Madrid, Spain; 2 INGEMM-IdIPAZ, Institute of Medical and Molecular Genetics, La Paz University Hospital, Madrid, Spain; 3 ITHACA, European Reference Network, Brussels, Belgium; 4 Dermatology Unit, La Paz University Hospital, Madrid, Spain; 5 Instituto de Investigaciones Biomedicas Sols-Morreale (IIBM), CSIC-UAM, Madrid, Spain

**Keywords:** palmoplantar keratoderma, *FAM83G*, genodermatosis, skin disorder, bilateral hearing loss, genomic medicine, massive paralleled sequencing

An incorrect **Funding** statement was provided. The funding information was incomplete. The correct **funding** statement reads:

“The author(s) declared that financial support was received for this work and/or its publication. This study has been funded by Instituto de Salud Carlos III through the projects PMP21/00063 and PMP22/00049 and by Next Generation EU funds, which finance the actions of the Recovery and Resilience Facility (RRF).”

The original version of this article has been updated.

